# Characterization and Uncertainty Assessment of a Certified Reference Material of Chloramphenicol in Methanol (GBW(E)082557)

**DOI:** 10.1155/2016/2348257

**Published:** 2016-07-13

**Authors:** Mengrui Yang, Min Wang, Jian Zhou, Yinqing Song, Tongtong Wang

**Affiliations:** Institute of Quality Standard and Testing Technology for Agro-Products, Chinese Academy of Agricultural Sciences, Beijing 100081, China

## Abstract

Prior to preparation of CRM candidate of chloramphenicol in methanol with a concentration of 100 mg/L, two independent methods including mass balance (MB) and quantitative nuclear magnetic resonance (qNMR) were employed to precisely measure the mass fraction of pure chloramphenicol materials. The mass fraction was assigned to be 99.8% with uncertainty of 0.3%. Homogeneity testing and stability study of chloramphenicol in methanol were examined by using high performance liquid chromatography. Additionally, the uncertainties originating from the process of CRM development were comprehensively evaluated. The experimental results indicate that the property value of this CRM is homogeneous and stable at 4°C for at least six months. The new CRM (GBW(E)082557) can be applicable to calibration of instrument and assurance of accuracy and comparability of results in routine measurement.

## 1. Introduction

A certified reference material (CRM), which is known as one kind of substance or material possessing one or more homogenous and stable property values with uncertainties under a specific confidence level, plays a very important role in the metrological sciences referring to instrument calibration, analytical method validation, and quantity transfer and traceability [1]. Therefore, precise and accurate assignment of property value should be a priority of all in the development of a CRM. To the pure organic substance, a series of measuring approaches are recognized and recommended by Bureau International des Poids et Mesures (BIPM). Mass balance (MB) method as an indirect purity measuring means is implemented by combination of several conventional instrumental measurements including chromatographic analysis of organic compounds, Karl-Fischer titration for water, inductively coupled plasma-mass spectrometry and atom absorbance spectroscopy for inorganic ions, and Thermal Gravity Analysis (TGA) for nonvolatile inorganic impurities [2–4]. In addition, differential scanning calorimetry (DSC) on the basis of the melting point depression caused by impurities is usually applicable to accurate purity measurement, while the impurities are soluble in the melting status and the true purity of main component is more than 98.5% [5–7]. Recently, quantitative nuclear magnetic resonance (qNMR) as a rising technique was proposed by BIPM for purity assay, which can be directly determined by using ideal internal standard. The qNMR approach is frequently applied in the metrology field and can be regarded as a primary method [8]. Therefore, it becomes more and more popular in purity measurement of organic compounds [9–12]. However, the selection of an internal standard is important and critical in the purity measurement. First of all, the selected internal standard must be a CRM to assure the traceability of measurement result to SI unit. Secondly, it must be soluble in common solvent and have no interference peaks with the target substance. Additionally, it is very important that the pointed signal peak of main component should be suitable and have no interference coming from the structurally related organic impurities and the internal standard.

Chloramphenicol, which was a common and famous antibiotic drug, has already been forbidden in the breeding process of livestock and poultry, because the drug residue in agroproducts could lead to toxic side effect to human body through food chain. Therefore, a series of testing standards for determination of chloramphenicol residue in honey, animal products, and animal feed were issued. Meanwhile, chloramphenicol CRMs have been shown to be important and essential in the implementation of these testing standards. Although various commercial CRMs associated with chloramphenicol are available according to the information from COMAR database (international database for certified reference materials), such as purity CRM of chloramphenicol from NIM (National Institute of Metrology, China) and matrix CRMs of chloramphenicol in pork and in honey from ERMM, diversity of chloramphenicol CRMs not only are enrichment for CRM database but also provide more selectable and suitable CRMs for routine work in testing laboratories. Prior to CRM development, a survey of utilization of chloramphenicol CRM in the testing standards was carried out. It was found that the calibration solution was usually prepared individually in each test laboratory by dissolving pure chloramphenicol CRM into methanol or acetonitrile with a concentration of ~100 mg/L. To reduce the operational steps and minimize the error from preparation of calibration solution in each laboratory, a user-friendly and timesaving CRM of chloramphenicol in methanol would be welcomed.

In this paper, the mass fraction of chloramphenicol material was precisely determined by using qNMR approach. To confirm the measurement result, MB as another independent method was carried out on purity determination. As mass fraction of chloramphenicol was accurately assigned, CRM of chloramphenicol in methanol was first developed strictly according to ISO Guide 34 and 35 and JJF 1006-1994 (Chinese Technical Norm of Primary Reference Material) [13–15]. Additionally, details on establishment of qNMR and MB methods for mass fraction assignment of chloramphenicol, preparation of chloramphenicol in methanol, homogeneity test and stability study, and uncertainty evaluation were sufficiently described.

## 2. Materials and Methods

### 2.1. Materials

All the high purity organic solvents were purchased from Merk. Certified reference materials of ethyl paraben (GBW(E) 100064) with a certificate value of 99.7% as an internal standard for qNMR measurement and chloramphenicol (GBW(E)060907) with a certificate value of 99.8% were supplied by National Institute of Metrology (NIM), China. Methanol-D_4_ was obtained from Sigma-Aldrich. The powder of chloramphenicol material with a labeled purity of 99.8% was purchased from Tokyo Chemical Industry (TCI) Co., Ltd., Japan.

### 2.2. Apparatus

A high performance liquid chromatography system (Shimadzu, Japan) equipped with Agilent Zorbax XDB-C18 (250 mm × 4.6 mm, 5.0 *μ*m) column and UV detector was applied in homogeneity test, stability study, and measurement of main component and unknown organic impurities. The Karl Fischer titration (METTLER TOLEDO DL39) was used for measurement of water content in chloramphenicol raw material. The analysis of nonvolatile impurities and volatile organic impurities was carried out on Thermal Gravity Analysis (PerkinElmer Pyris1, US) and gas chromatography coupled with flame ionization detector (GC-FID), respectively. A Bruker Avance III spectrometer with a cryoprobe at 400 MHz was employed to measure the mass fraction of chloramphenicol. All samples were weighed by using a Mettler AL104 analytical balance with a metrological verification certificate. Qualitative analysis was performed on a Bruker VERTEX 70 Infrared spectroscopy and Agilent mass spectrometry, respectively.

### 2.3. Purity Determination of Chloramphenicol

#### 2.3.1. Mass Balance Methods

The purity determination using mass balance method can be described with the following equation:

(1) $$\begin{array}{c} {P_{MB}=P}_{0}\left(\;100\%-X_{w}-X_{n}-X_{v}\;\right)\times 100\%, \end{array}$$

where *P*_0_ is main content determined by using HPLC area normalization method and *X*_w_ is the mass fraction of water; *X*_v_ is the mass fraction of volatile impurities; and *X*_n_ is the mass fraction of nonvolatile inorganic impurities.

The main contents and organic impurities of chloramphenicol were determined by using HPLC area normalization method, in which an Agilent Zorbax XDB-C18 (250 × 4.6 mm, 5 *μ*m) column was used and UV detector was set at 280 nm wavelength. The mobile phase was consisted of methanol and water adjusted with 0.2% acetic acid at a ratio of 40 : 60. The injection volume and the column oven temperature were 10 *μ*L and 30°C, respectively. In addition, the mass fraction of water (*X*_w_), volatile impurities (*X*_v_), and nonvolatile inorganic impurities (*X*_n_) were measured using a Karl Fischer titration, the headspace gas chromatography combined with FID detector, and the Thermal Gravity Analysis, respectively.

#### 2.3.2. Quantitative Nuclear Magnetic Resonance

The purity of chloramphenicol (*P*_NMR_) measured by qNMR can be calculated by the following equation:

(2) $$\begin{array}{c} P_{NMR}=\frac{I_{x}}{I_{std}}\cdot \frac{n_{std}}{n_{x}}\cdot \frac{M_{x}}{M_{std}}\cdot \frac{m_{std}}{m_{x}}\cdot P_{std}, \end{array}$$

where *I*_*x*_ and *I*_std_ are integrated peak area of chloramphenicol and internal standard, respectively; *n*_std_ and *n*_*x*_ are spin numbers of internal standard and chloramphenicol, respectively; *M*_*x*_ and *M*_std_ are molecular weights of chloramphenicol and internal standard, respectively; *m*_std_ and *m*_*x*_ are mass of internal standard and chloramphenicol in a determined sample. *P*_std_ is purity of internal standard.

The purity measurement was performed on Bruker Avance III 400 MHz ^1^H qNMR spectrometer which was equipped with pulsed field gradient probe at temperature of 293.4 K. The setting parameters are as follows: the pulse width of 3.98 s at 90° spectral band width of 8223.43 Hz, delay time of 60 s, and the number of transitions of 16. A sample including 10 mg ethyl paraben CRM as internal standard and 20 mg chloramphenicol was dissolved in 0.75 mL methanol-D_4_ solvent and was analyzed.

Acquisition on Bruker instrument was based on the command GO. During the NMR acquisition period, a steady-state pulse was applied prior to the relaxation decay. Sixteen dummy scans were acquired before data corrections. The receiver gain was automatically set by instrument. Each sample took about 10 min to finish. Seven samples were measured under the same conditions. All qNMR spectra were analyzed by using MestReNova software. A decay signal was automatically processed by Fourier transformation. The chemical shift of all spectra was referenced to residual ^1^H signals in deuterated solvent at 3.31 ppm for CD_3_OD. Phase correction and baseline correction were automatically applied according to the procedure of software guide. Peak integrations were set to an identical range for each spectrum.

### 2.4. Preparation of the CRM

According to the determined purity, the CRM of chloramphenicol in methanol was prepared by dissolving precisely weighted 100 mg chloramphenicol into 1000 mL methanol under strictly controlled temperature and moisture. After sufficient mixing and dissolution, the CRM of chloramphenicol in methanol was dispensed into ampoule bottles with 1.0 mL per each. A batch of CRM candidates including about 1000 units was produced and was stored at 4°C condition.

### 2.5. Homogeneity and Stability Test

Homogeneity and stability test was carried out by using HPLC-UV approach. 25 bottles were randomly selected from the batch and were examined to test the between-bottle homogeneity of chloramphenicol in methanol. Each bottle was measured in triplicate for testing within-bottle homogeneity. The homogeneity testing results were statistically analyzed by one-way analysis of variance (ANOVA).

The short-term and long-term stability testing was evaluated in eight days and six months, respectively. For short-term stability, the CRM bottles were exposed at 20°C and 40°C on predetermined days of 2, 4, 6, and 8; for long-term stability, the CRM bottles stored at 4°C conditions were analyzed in the 1st, 3rd, and 6th months. A new chloramphenicol calibration solution was freshly prepared when the stability was tested. At each time, a calibration solution and two bottles of CRM were analyzed by HPLC-UV method. The data of stability testing was assessed by regression analysis.

## 3. Results and Discussion

### 3.1. Purity Determination

**Figure 1:**
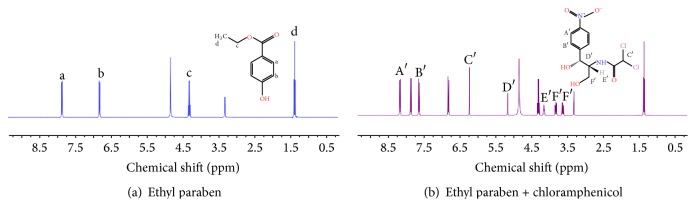
The H-NMR spectra of (a) internal standard (ethyl paraben and methanol-D_4_) and (b) sample solution (chloramphenicol, ethyl paraben, and methanol-D_4_).

**Figure 2:**
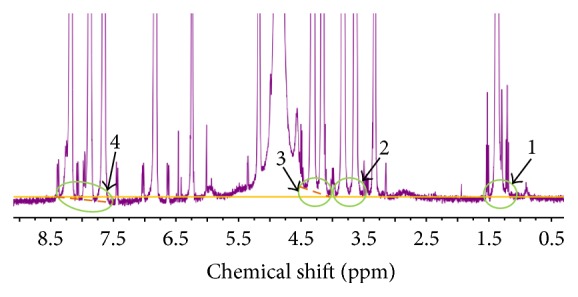
Baseline enlarged H-NMR spectrum of sample solution (chloramphenicol, ethyl paraben, and methanol-D_4_).

Quantitative nuclear magnetic resonance (qNMR) as a characterization approach for purity measurement was recently recommended by BIPM. To obtain precise and accurate results of purity, several principle rules should be noticed in the measurement processes. Measurement conditions including phase correction, baseline correction, pulse intervals, and spectral window were systematically optimized. First, a certified reference material as an internal standard is necessary to realize the traceability from testing result to SI unit. Second, the selected internal standard and target analyte should be simultaneously soluble in the same and common deuterium labeled solvent. Third, the pointed signal peaks for quantification must have no interference for each other. Ethyl paraben CRM was commonly thought of as an ideal internal standard for purity measurement in many researches; thus, the ethyl paraben was selected in this study. In comparison with spectrum of only ethyl paraben in Figure 1(a), H peaks ascribed to internal standard and analyte can be easily identified in Figure 1(b) which shows the H-NMR spectrum of mixture of ethyl paraben and chloramphenicol in methanol-D_4_. As shown in Table 1, three pairs of peaks at different chemical shifts with no overlap were chosen for quantitative analysis of chloramphenicol; however, different purity results ranging from 98.94% to 99.82% were obtained according to the equation in Section 2.3.2. To judge which result is more accurate and reliable, the baseline of NMR spectrum was zoomed up as shown in Figure 2. It is found that only the two peaks in pair A are in the same level of baseline as shown in cycle 1 and cycle 2. However, there are additional tiny peaks in cycles 1, 3, and 4, which are considered to be from the structurally related organic impurity by comparing with spectrum of internal standard only. Due to the inevitable interference from impurities, a commercial chloramphenicol CRM was used for checking the impurities and evaluating its influence on value assignment. Although the similar structurally related organic impurity was also observed, its contribution to peak integration in qNMR can be ignored. In this case, it was presumed that selection of quantitative peaks on the basis of same baseline level might be more critical in this study. Therefore, the purity of 99.82% was considered to be the closest to the true value among the three results.

**Figure 3:**
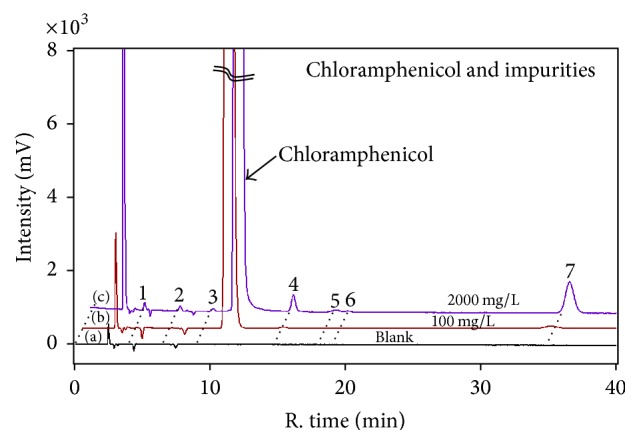
Chromatography of (a) methanol blank, (b) 100 mg/L chloramphenicol in methanol, and (c) 2000 mg/L chloramphenicol in methanol.

For further confirmation, mass balance (MB) method was carried out on the measurement of purity. On the contrary with qNMR, MB is an indirect purity measurement method and is accomplished by precise measurements of impurities including organic impurities, moisture, and volatile and nonvolatile impurities. The analysis of organic impurities is generally measured by using chromatography. In this research, HPLC-UV was performed to analyze organic impurities in chloramphenicol. In order to identify and measure impurities sufficiently and comprehensively, methanol (blank) and chloramphenicol in methanol with concentrations of 100 mg/L and 2000 mg/L were determined on the UV absorbance at 280 nm. In comparison with methanol blank, peaks of chloramphenicol and impurities were observed and separated in Figure 3. A total of seven impurities peaks were detected at the concentration of 2000 mg/L, while two peaks (impurity 4 and impurity 7) were obviously observed at a concentration of 100 mg/L as shown in Figures 3(b) and 3(c). It should be emphasized that the concentration for the purity measurement by using HPLC area normalization method must be prepared as high as possible to guarantee signal response of all impurities sufficiently. Therefore, the purity determined by HPLC-UV was 99.84% with a standard deviation of 0.02% shown in Table 2. For the water measurement, a Karl-Fischer coulometric titrator was used. Mass fraction of water was determined and was calculated to be 0.025% with standard deviation of 0.003% as shown in Table 2. In this study, the nonvolatile inorganic impurities were measured by heating the samples from 35°C to 600°C at 10°C/min. Gradual evaporation process curve was obtained and the weight of sample did not change by heating up to 600°C. The volatile organic impurities were determined by GC-FID. The headspace gas chromatography combined with FID detector was carried out on a column of DB-624 (30 m × 0.32 mm, 1.8 *μ*m) to determine volatile impurities associated with twelve kinds of conventional solvents including methanol and acetonitrile. Parameters were set as follows: hydrogen, 40 mL/min, air, 400 mL/min, transfer line temperature, 100°C, and injection volume, 50 *μ*L. The initial temperature was 45°C and it was held for 5 min. The temperature was increased to 120°C at 7°C/min and to 230°C at 15°C/min and was held for 8 min. It was found that several peaks with intensity less than 3 times signal-to-noise ratio (LOD) were detected. Therefore, the mass fraction of nonvolatile inorganic and volatile organic impurities can be ignored.

### 3.2. Homogeneity Test

For the homogeneity test, 25 ampoule bottles of samples were chosen from the batch of CRMs randomly and were determined in triplicates for each by using optimized HPLC-UV method mentioned in the above section. The area of signal peaks was recorded by HPLC-UV area normalization method as shown in Table 3. According to General and Statistical Principles for Characterization of Reference Material JJF 1343-20012, the data was analyzed by one-way analysis of variance (ANOVA) and the results were shown in Table 4. It was found that the calculated *F* value was less than the critical value of *F*_0.05_(24,50). It has been demonstrated that the homogeneity of chloramphenicol in methanol was homogenous under the minimum sampling of 10 *μ*L. 

### 3.3. Stability Study

#### 3.3.1. Short-Term Stability Study

To assess the effect of temperature change in delivery, portions of samples were prestored at 20°C and 40°C to simulate the shipment conditions. Two ampoule bottles of sample were stored at each temperature for 2, 4, 6, and 8 days. After the indicated storage periods, the samples and a freshly prepared chloramphenicol in methanol as calibration solution were determined in triplicate using the HPLC-UV method. The stability data at 20°C and 40°C were plotted against time, respectively, and the regression lines were calculated (shown in Table 5). The observed slope *b*_1_ was tested for significance using* t*-test, with *s*(*b*_1_) being the uncertainty of slope and *t*_0.95,*n*−2_ of critical *t* value for a confidence level of 95% and *n* − 2 degree of freedom. As shown in Table 3, no significance slope was found for storage temperatures of 20°C (|*b*_1_| < *t*_0.95,*n*−2_ · *s*(*b*_1_)), whereas the slope was highly significant for a storage temperature of 40°C (|*b*_1_| > *t*_0.95,*n*−2_ · *s*(*b*_1_)). This led to a conclusion that if the shipment temperature was not higher than 20°C, the uncertainty of short-term stability at 40°C can be considered to be negligible.

#### 3.3.2. Long-Term Stability Study

To satisfy the requirement of CRMs, the chloramphenicol in methanol, which was stored at 4°C, was measured at 1st, 3rd, and 6th months. Long-term stability of chloramphenicol in methanol was examined by using the same analytical method and statistical analysis approach as described in short-term stability study. As shown in Table 6, no significance slope was found. This indicates that chloramphenicol in methanol is stable for at least six months at 4°C.

### 3.4. Evaluation of Uncertainties

**Figure 4:**
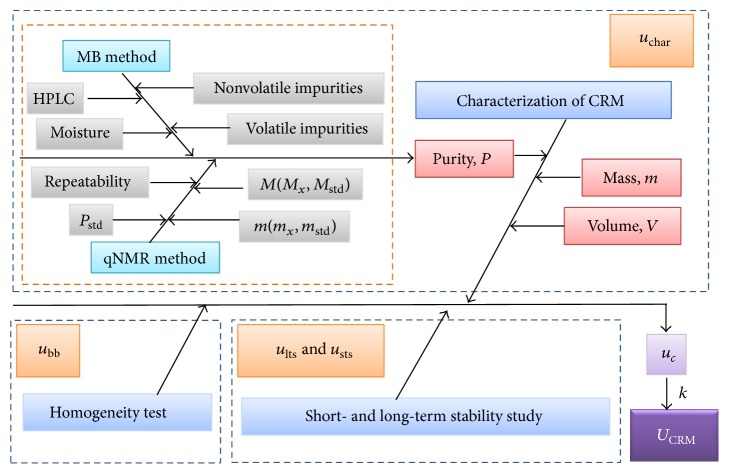
Uncertainty sources in the development process of chloramphenicol in methanol CRM.

In strict accordance with General and Statistical Principles for Characterization of Reference Materials JJF 1343-2012 [16], the sources of uncertainties of chloramphenicol in methanol CRM can be classified into three main parts (see Figure 4): uncertainty from homogeneity test *u*_bb_, uncertainties from long-term and short-term stability *u*_lts_ and *u*_sts_, and uncertainty from characterization *u*_char_. Meantime, *u*_char_ was comprised of the following items: balance precision, volume variation of volumetric flask, and purity of chloramphenicol material in which the uncertainty of purity was evaluated through combining uncertainties originating from purity determination process by two independent methods of mass balance and qNMR as shown in the orange dashed line rectangle in Figure 4.

For MB method, the standard uncertainty *u*(*P*_MB_) can be calculated by the following equation:

(3) uPMB=PMBuP0P02+u2Xw+u2Xn+u2Xv1−Xw−Xn−Xv2,

in which *u*(*P*_0_) = *u*_HPLC_ can be evaluated by using the following equation:

(4) $$\begin{array}{c} u_{HPLC}=\sqrt{u_{1}^{2}+u_{2}^{2}}, \end{array}$$

where *u*_1_ is uncertainty originating from the standard deviation of HPLC-UV measurement; *u*_2_ is uncertainty from difference of UV response factors of impurities, which can be estimated by the following equation:

(5) $$\begin{array}{c} u_{2}=\frac{\sqrt{\sum_{i=1}^{n}u_{2-i}^{2}}}{\sqrt{3}}=\frac{\sqrt{\sum_{i=1}^{n}{\left(B_{i\max\lambda }-B_{i\;\;value\;\;\lambda }\right)}^{2}}}{\sqrt{3}}, \end{array}$$

where *B*_*i*max⁡*λ*_ is the maximum percentage of impurity *i* at specific wavelength; *B*_*i*  value  *λ*_ is the percentage of impurity *i* at characterization wavelength; *u*_2−*i*_ is uncertainty of impurity *i*. In this study, the wavelengths are selected at 210 nm, 235 nm, 254 nm, 280 nm, and 300 nm.

Additionally, uncertainty of moisture measurement *u*(*X*_w_) can be expanded by using the mean of measurements, respectively. In this case, the property value is more reliable and reasonable under the technical limitation. In addition, uncertainties of *u*(*X*_n_) and *u*(*X*_v_) can be ignored.

For qNMR, the standard uncertainty *u*(*P*_NMR_) can be calculated by the following equation:

(6) $$\begin{array}{c} u\left(\;P_{NMR}\;\right)=P_{NMR}\sqrt{{\left(\frac{u\left(I_{x}/I_{std}\right)}{I_{x}/I_{std}}\right)}^{2}+{\left(\frac{u\left(M_{x}\right)}{M_{x}}\right)}^{2}+{\left(\frac{u\left(M_{std}\right)}{M_{std}}\right)}^{2}+{\left(\frac{u\left(m_{x}\right)}{m_{x}}\right)}^{2}+{\left(\frac{u\left(m_{std}\right)}{m_{std}}\right)}^{2}+{\left(\frac{u\left(P_{std}\right)}{P_{std}}\right)}^{2}}, \end{array}$$

where *u*(*I*_*x*_/*I*_std_) was expressed by the relative standard deviation of measurement; *u*(*m*_std_) and *u*(*m*_*x*_) were from the balance weight; *u*(*P*_std_) was obtained from the certificate of ethyl paraben CRM (GBW(E)100064); *u*(*M*_*x*_) and *u*(*M*_std_) were calculated by the following equation:

(7) $$\begin{array}{c} u\left(\;M\;\right)=\sqrt{\overset{n}{\underset{j=1}{\sum }}{\left[N_{j}u_{j}\right]}^{2}}, \end{array}$$

where *N*_*j*_ is atom number of *j* element; *u*_*j*_ is uncertainty of relative atomic mass of *j* element which was cited from IUPAC table of atom weights of elements.

Therefore, uncertainty of purity measurement was evaluated as follows:

(8) $$\begin{array}{c} u\left(\;P\;\right)=\sqrt{{u{\left(P_{MB}\right)}^{2}+u\left(P_{NMR}\right)}^{2}}. \end{array}$$

As we mentioned above, chloramphenicol in methanol was prepared by dissolving chloramphenicol into methanol. Thus, the concentration of CRM solution can be calculated by the following equation:

(9) $$\begin{array}{c} c=\frac{1000mP}{V}, \end{array}$$

where *c* is the concentration of chloramphenicol in methanol (mg/L); *m* is amount of chloramphenicol (mg); *P* is the purity of chloramphenicol; *V* is volume of methanol (mL). The relative uncertainty of characterization can be expressed by the following equation:

(10) uchar,relucc=uPP2+umm2+uVV2.

According to above analysis of uncertainty, the evaluated uncertainty results are summarized in Table 7.

### 3.5. Value Verification

In order to check up the reliability and accuracy of the assigned value of chloramphenicol in methanol CRM in this development project, an existing certified reference material of chloramphenicol (GBW(E)060907) was selected as calibrator to measure the property value of chloramphenicol in methanol CRM. A calibration solution of chloramphenicol with a concentration of 100.9 mg/L, which is approximate to the assigned value of (100 ± 2) mg/L, was precisely prepared by verified balance and volumetric flask. The developed CRM of chloramphenicol in methanol as an unknown sample was measured in triplicate. As shown in Table 8, the mean of measured value is 99.7 mg/L with a standard deviation of 0.2 mg/L. It was found that the measured value was covered by the assigned value accurately. Therefore, it can be approved that the assigned value of chloramphenicol in methanol was reliable and accurate.

## 4. Conclusion

A user-friendly and time-saving CRM of chloramphenicol in methanol (GBW(E)082557) was successfully developed and was approved by General Administration of Quality Supervision, Inspection, and Quarantine of China. In the study, the qNMR and mass balance methods were employed in the precise measurement of chloramphenicol purity. The homogeneity, stability, and uncertainty of this CRM were sufficiently studied according to the technical specification. In addition, the selection of proton peak in qNMR spectrum for purity measurement was comparatively studied.

## Figures and Tables

**Table 1:** Mass fraction of chloramphenicol calculated with different pointed signal peaks (*n* = 7).

Pairs	Peak of *I*_*x*_/ppm	Peak of *I*_std_/ppm	Mean of *P*_NMR_/%	SD/%
A	d	F′	99.82	0.07
B	c	F′	99.13	0.09
C	a	A′	98.94	0.05

“*n*” means different data sets measured under the same conditions.

**Table 2:** Mass fraction of chloramphenicol measured by MB methods.

Sample vials number	HPLC/%	Moisture/%	Purity/%
1	99.87	0.020	99.85
2	99.84	0.030	99.81
3	99.84	0.026	99.81
4	99.87	0.025	99.85
5	99.83	0.028	99.80
6	99.83	0.023	99.81
7	99.82	0.024	99.80
Mean	99.84	0.025	99.82
SD	0.020	0.003	0.02

**Table 3:** Data of homogeneity study.

Vials number	Peak area				
	1	2	3	Mean	SD
1	3377539	3455815	3426396	3419917	39538
2	3363161	3383494	3413952	3386869	25563
3	3373464	3438840	3444717	3419007	39551
4	3361335	3400715	3431809	3397953	35318
5	3361386	3385011	3400994	3382464	19926
6	3370750	3440166	3441286	3417401	40405
7	3396404	3442398	3445094	3427965	27366
8	3412038	3456559	3452226	3440274	24549
9	3492616	3414137	3400516	3435756	49711
10	3412637	3423430	3431176	3422414	9311
11	3382569	3410793	3415548	3402970	17827
12	3351945	3362416	3401855	3372072	26319
13	3359186	3400385	3432492	3397354	36747
14	3363789	3419854	3419894	3401179	32381
15	3373310	3417544	3432514	3407789	30784
16	3377488	3418379	3415865	3403911	22917
17	3382255	3412441	3413938	3402878	17876
18	3393284	3425055	3415432	3411257	16292
19	3406204	3444518	3444612	3431778	22148
20	3403316	3406961	3405709	3405329	1852
21	3364827	3408684	3409946	3394486	25693
22	3382216	3383257	3405014	3390162	12872
23	3400230	3387406	3414257	3400631	13430
24	3361051	3391923	3404463	3385812	22342
25	3353175	3403030	3394614	3383606	26688

**Table 4:** Results of homogeneity study.

Items	Results
Total mean	3405649
Total SD	28716
Between-bottle	946997904
Within-bottle	765842476
*F*	*F* = *s*_1_^2^/*s*_2_^2^ = 1.24
*F* critical value	*F*_0.05_(24,50) = 1.740
Evaluation result	*F* < *F*_0.05_(24,50)

**Table 5:** Result of short-term stability study.

Time/day	Value/(mg/L)	
	Condition/20°C	Condition/40°C
0	100.7	100.7
2	100.2	99.8
4	100.0	96.4
6	99.4	93.3
8	99.9	92.4

Mean	99.8	96.6

*b*_1_	−0.119	−1.153
*b*_0_	100.5	101.1
*s* ^2^	0.1053	0.7423
*s*(*b*_1_)	0.0513	0.1362
*t*_0.95,*n*−2_	3.18	3.18
Conclusion	|*b*_1_ | < *t*_0.95,*n*−2_ · *s*(*b*_1_), stable	|*b*_1_ | > *t*_0.95,*n*−2_ · *s*(*b*_1_), instable

**Table 6:** Results of long-term stability study.

Time/month	Value/(mg/L)			
	0	1	3	6
#1	100.4	100.4	101.4	100.2
	101.2	99.7	100.9	99.8
	101.1	99.6	101.0	100.5

#2	100.2	99.1	100.5	100.3
	101.6	101.4	100.3	99.9
	100.8	100.8	100.6	99.8

Mean	100.9	100.2	100.8	100.1

*b*_1_	−0.0845			
*b*_0_	100.7			
*s* ^2^	0.1801			
*s*(*b*_1_)	0.0926			
*t*_0.95,*n*−2_	4.3			
Conclusion	|*b*_1_ | < *t*_0.95,*n*−2_ · *s*(*b*_1_), stable			

**Table 7:** Results of uncertainty.

Items	Assessment and calculation	Results
*u*(*m*)	(1) variability of balance measurement (empirical estimation)	0.41%
	(2) uncertainty of balance calibration (metrological verification certificate)	

*u*(*V*)	(1) error of volumetric flask (metrological verification certificate)	0.35%
	(2) random error (empirical estimation)	
	(3) variation with temperature (calculation)	

*u*(*P*)_rel_	$u{\left(P\right)}_{rel}=\frac{u\left(P\right)}{P}=\sqrt{\frac{u{\left(P_{MB}\right)}^{2}}{P_{MB}}+\frac{u{\left(P_{NMR}\right)}^{2}}{P_{NMR}}}$	0.28%

*u*_char,rel_	$u_{char,rel}=\frac{u(c)}{c}=\sqrt{{\left[\frac{u\left(P\right)}{P}\right]}^{2}+{\left[\frac{u\left(m\right)}{m}\right]}^{2}+{\left[\frac{u\left(V\right)}{V}\right]}^{2}}$	0.61%

*u*_bb,rel_	$u_{bb,rel}=\frac{\sqrt{\left(s_{1}^{2}-s_{2}^{2}\right)/n}}{{\overset{-}{X}}_{bb}}$	0.23%

*u*_lts_ & *u*_sts_	$u_{lts,rel}=s\left(b_{1}\right)\cdot t/\overset{-}{X}$ & $u_{sts,rel}=s\left(b_{1}\right)\cdot t/\overset{-}{X}$	0.56% & 0.41%

*u*_*c*,rel_	$u_{c,rel}=\sqrt{u_{char,rel}^{2}+u_{bb,rel}^{2}+u_{lts,rel}^{2}+u_{sts,rel}^{2}}$	0.94%

*U*_CRM_	*U*_CRM_ = *k* · *u*_c_ = *k* · *c* · *u*_*c*,rel_; (*k* = 2, 95% confidence level)	2 mg/L

**Table 8:** Verification results of chloramphenicol in methanol CRM (unit: mg/L).

Item	Measured value					Assigned value
	1	2	3	Mean	SD	
CRM	99.5	99.9	99.7	99.7	0.2	100 ± 2
